# Hybrid Nanosystems Based on Nicotinate-Functionalized Mesoporous Silica and Silver Chloride Nanoparticles Loaded with Phenytoin for Preventing *Pseudomonas aeruginosa* Biofilm Development

**DOI:** 10.3390/ph15070884

**Published:** 2022-07-18

**Authors:** Maider Ugalde-Arbizu, John Jairo Aguilera-Correa, Aranzazu Mediero, Jaime Esteban, Paulina L. Páez, Eider San Sebastian, Santiago Gómez-Ruiz

**Affiliations:** 1 Departamento de Química Aplicada, Facultad de Química, Euskal Herriko Unibertsitatea (UPV/EHU), Paseo Manuel Lardizabal 3, 20018 San Sebastián, Spain; maider.ugalde@ehu.eus (M.U.-A.); eider.sansebastian@ehu.eus (E.S.S.); 2 Clinical Microbiology Department, IIS-Fundación Jiménez Diaz, UAM, Avenida Reyes Católicos 2, 28040 Madrid, Spain; jesteban@fjd.es; 3 COMET-NANO Group, Departamento de Biología y Geología, Física y Química Inorgánica, ESCET, Universidad Rey Juan Carlos, C/Tulipán s/n, 28933 Móstoles, Spain; 4 CIBER de Enfermedades Infecciosas (CIBERINFEC), 28029 Madrid, Spain; 5 Bone and Joint Unit, IIS-Fundación Jimenez Diaz, UAM, Avenida Reyes Católicos 2, 28040 Madrid, Spain; aranzazu.mediero@quironsalud.es; 6 Departamento de Ciencias Farmacéuticas, Facultad de Ciencias Químicas, Universidad Nacional de Córdoba, Unidad de Investigación y Desarrollo en Tecnología Farmacéutica (UNITEFA), Consejo Nacional de Investigaciones Científicas y Técnicas (CONICET), Haya de la Torre y Medina Allende, Ciudad Universitaria, Córdoba X5000HUA, Argentina; plpaez@unc.edu.ar

**Keywords:** MSN, silver chloride, phenytoin sodium, *P. aeruginosa*, biofilm, wound healing

## Abstract

*Pseudomonas aeruginosa* (PA) is one of the most common bacteria isolated from chronic wounds and burns. Its treatment is a challenge due to antimicrobial drug resistance and biofilm formation. In this context, this study aimed to perform the synthesis and full characterization of hybrid nanosystems based on mesoporous silica nanoparticles (MSNs) functionalized with a nicotinic ligand and silver chloride nanoparticles, both phenytoin sodium (Ph)-loaded and unloaded, to evaluate the antibacterial properties against three different strains of PA (including two clinical strains) in a planktonic state and as biofilms. Ph is a well-known proliferative agent, which was incorporated into the hybrid nanomaterials to obtain an effective material for tissue healing and prevention of infection caused by PA. The Ph-loaded materials promoted a quasi-complete inhibition of bacterial growth in wound-like medium and biofilm development, with values of 99% and 96%, respectively, with selectivity indices above the requirements for drugs to become promising agents for the topic preventive treatment of chronic wounds and burns.

## 1. Introduction

*Pseudomonas aeruginosa* is one of the most common bacterial species in chronic wounds, and it is known as a critical cause of mortality and morbidity in burnt patients [1]. In addition, *P. aeruginosa* is also known to have a propensity to form biofilms, thereby decreasing antibiotic treatment efficacy and resulting in more chronic infections and prolonged hospital stays. Therefore, the prevention and the treatment of infections caused by *P. aeruginosa* are currently a challenge [2,3].

In the last decade, nanomedicine has developed significantly in fields such as drug delivery. In this field, although there are different types of nanocarriers, mesoporous silica nanoparticles (MSNs) represent one of the most important and extensively used support materials. The main reason for this is that MSNs present notable properties such as a good degree of porosity, large surface area and pore volume, and ease of surface modification [4,5]. Thus, these nanoparticles have been studied, among others, as support for organic and inorganic compounds, proteins, metal nanoparticles, etc., constituting particularly good agents against bacterial infections [6,7].

In this work, conveniently functionalized and fully characterized MSNs were used as therapeutic agents for the prevention of bacterial biofilm development in chronic wounds and burns. The latter was achieved by decoration of the surface of MSNs with silver chloride nanoparticles. Silver-containing systems were used in the past for topical antibacterial treatments of burns and wounds; for example, silver sulfadiazine was introduced in the 1970s as an antibacterial agent [8]. In the same context, MSNs have previously been applied as a support of silver nanoparticles against several bacteria [9,10,11]. However, the use of MSN as a support for silver chloride nanoparticles is still very limited [12,13] and has not yet been studied against the bacterium *P. aeruginosa*.

AgCl NPs are generally attached to MSNs through coupling agents such as 3-mercaptopropyltrimethoxysilane or 3-aminopropyltriethoxysilane [14,15]. However, in this work, in situ generated nicotinamide was used as the coupling agent. Such a novel coupling group was designed with the aim of potentially increasing the antimicrobial activity of the synthesized nanocarriers, since several nicotinamides have been described to show some antimicrobial activity [16,17,18]. Actually, the bactericidal effect of this hybrid nanosystem was analyzed in vitro, showing excellent and promising results in the inhibition of wound-like medium and biofilm development.

Another key aspect in microbial infections is the development of effective materials that not only prevent infections caused by PA but also promote tissue healing; the latter was successfully achieved in this work by the adsorption on the functionalized mesoporous material of the well-known proliferative agent phenytoin sodium [19,20,21], which was loaded in the system and showed an additive effect within all the other components of the synthesized nanomaterials.

## 2. Results

### 2.1. Synthesis and Physicochemical Characterization of Functionalized NPs

**Scheme 1:**
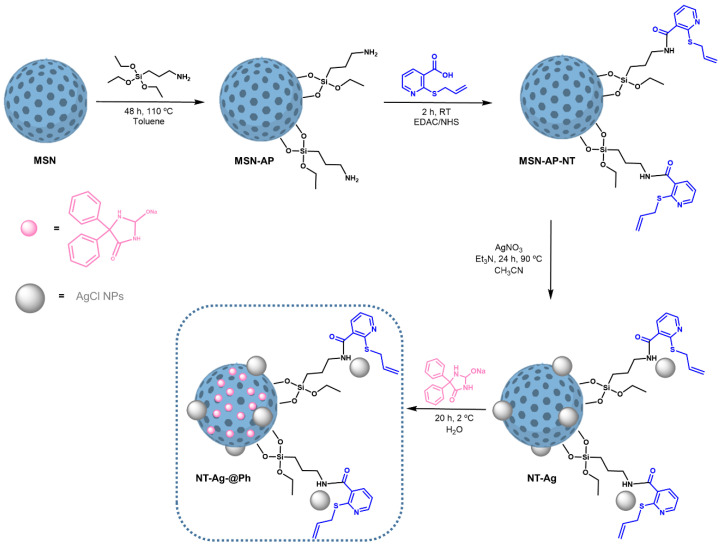
Synthesis of the materials.

MSN were functionalized with 3-aminopropyltriethoxysilane to give **MSN-AP** (Scheme 1). Subsequently, these amino-functionalized materials were also treated with 10% 2-allythionicotinic acid using an EDAC/NHS coupling reaction to give **MSN-AP-NT** (Scheme 1). After functionalization with NT, the formation of AgCl nanoparticles was achieved by the reaction of the material with AgNO_3_ under reflux and darkness, to obtain the **NT-Ag** material (Scheme 1). Finally, the **NT-Ag** material was loaded with phenytoin sodium in water and at low temperature to achieve the final material **NT-Ag@Ph** (Scheme 1).

#### 2.1.1. Analysis of Size, Morphology, and Textural Properties

**Figure 1:**
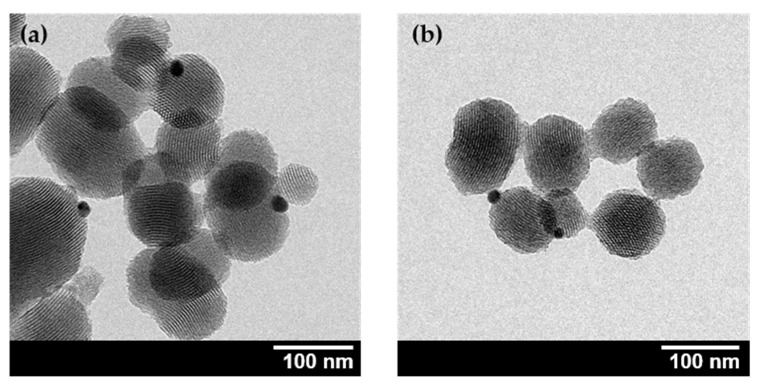
TEM images of **NT-Ag** (**a**) and **NT-Ag@Ph** (**b**).

The final materials were characterized by transmission electron microscopy (TEM) (Figure 1). The micrographs were analyzed using ImageJ^®^ software in order to determine the morphology and particle size. As can be seen in Figure 1, the nanoparticles showed an oval morphology with pores crossing the particles. The nanoparticles had a mean size of 97.23 ± 19.75 nm and 95.08 ± 15.93 nm, for **NT-Ag** and **NT-Ag@Ph**, respectively (for particle size distributions, see Figure S1 of SI). Moreover, as can be observed in Figure 1, the morphology and particle sizes were similar between the final materials; thus, it can be deduced that the encapsulation in the porous system of phenytoin sodium did not influence in the morphology and the particle size of the silica nanoparticles. Lastly, spherical nanoparticles were observed adhered to MSNs with an average size of 15–20 nm that could be compatible with Ag nanoparticles.

The characterization of the textural properties, such as surface area (BET) and pore volume and diameter, were estimated for both the starting and the final materials by means of the analysis of nitrogen adsorption–desorption isotherms (see Figure S2 of SI). Textural property data derived from such isotherms are collected in Table 1. The starting bare nanoparticles (MSN) showed a typically high BET surface area and pore volume of 1102 m^2^/g and 0.75 cm^3^/g, respectively.

These parameters decreased importantly in both the unloaded (**NT-Ag**) and the phenytoin-loaded (**NT-Ag@Ph**) nicotinamide-functionalized materials, with the BET surface area being up to nine times smaller for **NT-Ag** (115 m^2^/g) and almost 23 times smaller for **NT-Ag@Ph** (48 m^2^/g). This was indicative of a high degree of functionalization of MSN, as well as of the encapsulation of a large amount of Ph in the pores. On the basis of the literature, we hypothesize that the Si–OH terminal groups present in the tunnel walls of MSN may have been interacting with the Ph molecules via intermolecular forces such as hydrogen bonds [22]. Ph encapsulation inside the pores was also reflected in the significant decrease in the pore volume and pore size of the materials. Thus, pore volume decreased up to six times for unloaded material **NT-Ag** (0.18 cm^3^/g) and 10 times for loaded material **NT-Ag@Ph** (0.07 cm^3^/g) compared with the unmodified MSNs, whereas pore size decreased from ca. 2.84 nm in **MSN** to less than 2.00 nm in **NT-Ag** and **NT-Ag@Ph**.

#### 2.1.2. Quantification of the Functionalization Degree by Thermogravimetry and Inductively Coupled Plasma Atomic Emission Spectroscopy

**NT-Ag** and **NT-Ag@Ph**, as well as the initial and intermediate materials (**MSN**, **MSN-AP**, and **MSN-AP-NT**) were characterized by TGA to determine the quantity of AP, nicotinate, and phenytoin sodium that had been incorporated in the MSNs. The thermograms (Figure S3 in SI) showed a clear mass loss between 120 and 650 °C; in this context, the mass losses increased with consecutive functionalization reactions. Detailed analysis of the thermograms revealed the functionalization ratios shown in Table 2. The initial material (**MSN**) was subjected to the coupling of AP and, subsequently, of NT and yielded **MSN-AP-NT** with ca. 8.7% AP and around 2.5% in weight, translating into 0.14 mmol of NT per gram of MSNs. The silver content in the silver-loaded material (**NT-Ag**) estimated by ICP-AES revealed that the amount of silver incorporated was 0.03 mmol Ag per gram of MSNs. Finally, the phenytoin encapsulation process yielded **NT-Ag@Ph** with ca. 9.3% Ph in weight, which translates into 0.37 mmol of Ph per gram of MSNs.

#### 2.1.3. Characterization by Powder X-ray Diffraction Studies

**Figure 2:**
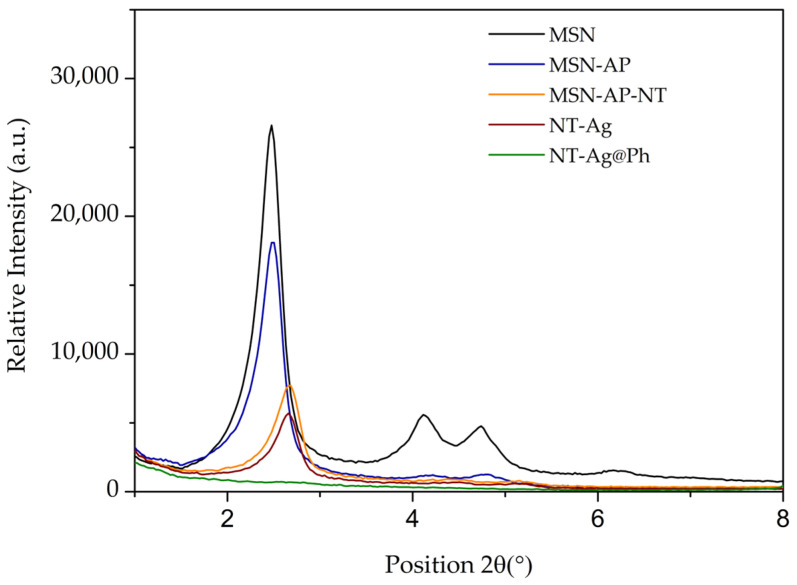
Small-angle XRD patterns of all materials.

The starting material **MSN** and the corresponding functionalized materials were characterized by powder X-ray diffraction (Figure 2 and Table S1). All materials showed a high-intensity diffraction peak at a 2θ of ca. 2.47° assigned to the (100) Miller plane, which confirmed the mesoscopic order of the materials attributed to their hexagonal porous structure promoted by the use of CTAB as surfactant. Additionally, two signals were observed around 2θ 4.1° and 4.7°, corresponding to the (110) and (200) Miller planes, respectively. The intensity of the (100) peak decreased after the different functionalization reactions due to the partial blocking of the scattering points of the porous system caused by the incorporation of the organic and/or metallic fragments in the system [23]. Furthermore, the right shift of the signal at 2.47° upon functionalization with NT and incorporation of Ag was indicative of a slight decrease in the pore size [24,25]. In addition, as a consequence of the efficient functionalization, in the modified systems, a quenching of signals at 2θ values of 4.1° and 4.7° (which appeared in the unmodified MSN) was observed in the functionalized systems. In addition, the presence of AgCl nanoparticles and the correct encapsulation of Ph in the pores of MSN were confirmed by large-angle diffractograms, (Figure S4 in SI) of **NT-Ag** and **NT-Ag@Ph**.

The successful functionalization and Ph encapsulation of the materials were further confirmed by FT-IR, diffuse reflectance UV–visible spectroscopy, and solid-state NMR spectroscopic studies (see details in SI, Figures S5–S7).

### 2.2. In Vitro Studies of Antibacterial Activity

#### 2.2.1. Minimum Inhibitory Concentration (MIC) and Minimal Bactericidal Concentration (MBC)

The antibacterial effects of the unloaded and phenytoin sodium (Ph)-loaded materials were evaluated by studying the MIC and MBC values upon incubation of three different planktonic *P. aeruginosa* strains (ATCC27853 and the clinical patient-derived PA8 and PA13 strains) with different concentrations of Ph-loaded and unloaded materials.

Results, collected in Table 3, revealed that the Ph-unloaded **NT-Ag** displayed MIC values of 250, 62.50, and 31.25 µg/mL for ATCC27853, PA8, and PA13 strains, respectively. On the other hand, MBC values were estimated to be 500, 125, and 250 µg/mL for ATCC27853, PA8, and PA13 strains, respectively. Interestingly, the encapsulation of Ph in **NT-Ag@Ph** promoted a decrease in the MIC values in the first two strains to 31.25 µg/mL but had no effect on the MBC values. The latter result was somewhat unexpected, since Ph is only known as a wound-healing agent, and its antibacterial activity was surprising in the context of the studied materials.

It is important to note that the release of Ph in physiologic medium was studied for material **NT-Ag@Ph** observing that already after only 2 h more than 50% of the loaded Ph is already in the solution (with almost 60% already in suspension after 48 h, see Figure S8 of SI) indicating that the effect of Ph is patent from the beginning of the experiments

Trying to look for a potential synergistic effect between AgCl nanoparticles and Ph, viability studies of *P. aeruginosa* in planktonic form were carried out in the presence of different concentrations of co-administered substances (see next Section 2.2.2).

#### 2.2.2. Effect of Coadministration of Silver Nitrate and Phenytoin Sodium

**Figure 3:**
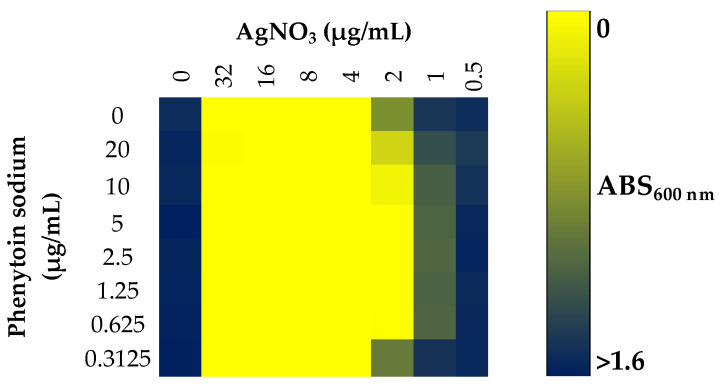
Synergy between silver nitrate and phenytoin sodium against planktonic state of ATCC27853. Blue indicates bacterial growth, while yellow indicates the absence of bacterial growth.

The possible synergistic, antagonistic, or indifferent activity of the combined administration of silver and phenytoin sodium toward the viability of the planktonic form of *P. aeruginosa* was studied in ATCC27853 strain, using the checkerboard methodology [26,27]. The degree of interaction between silver nitrate and phenytoin sodium was quantified by the fractional inhibitory concentration (FIC). Results shown in Figure 3, revealed that, as expected, Ph at any concentration between 0 and ca 0.31 µg/mL had no antibacterial activity, whereas silver nitrate showed antibacterial activity at concentrations above 2 µg/mL. On the other hand, the FIC index for both agents was found to be 0.75, indicative of an additive effect, i.e., they were additive agents in terms of antibacterial properties (see Section 4).

#### 2.2.3. Minimal Biofilm Inhibitory Concentration (MBIC) and Minimal Biofilm Eradication Concentration (MBEC)

The antibiofilm effect of **NT-Ag** and **NT-Ag@Ph** materials was evaluated by studying the MBICs and MBECs (Table 4). Different concentrations of **NT Ag** and **NT-Ag@Ph** were incubated with three different *P. aeruginosa* biofilms. As shown in Table 4, the encapsulation of Ph in **NT-Ag** did not significantly modify the MBIC and MBEC values. In this sense, identical MBIC values of 125 and 500 µg/mL were obtained for strains PA8 and PA13 when using **NT-Ag** and **NT-Ag@Ph**, observing that a decrease from 250 to 125 µg/mL in the ATCC27853 strain was the unique activity difference of the complete analysis. MBEC values were >1000 µg/mL for both materials and all strains, except the MBEC value for **NT-Ag@Ph** incubated with strain PA8, where the MBEC was 1000 µg/mL.

#### 2.2.4. Effect on Biofilm Development

**Figure 4:**
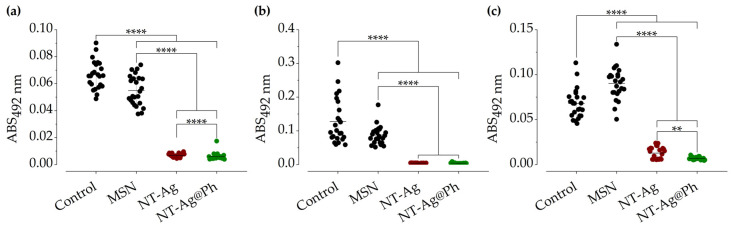
Inhibition of biofilm development of ATCC27853 (**a**), PA8 (**b**), and PA13 (**c**) in the presence of **MSN** (as a control, black), **NT-Ag** (garnet), and **NT-Ag@Ph** (green) at 125 µg/mL. ** *p* < 0.01, **** *p* < 0.0001 according to Mann–Whitney and Kruskal–Wallis tests.

The effect of **NT-Ag** and **NT-Ag@Ph** on *P. aeruginosa* biofilm development was studied using an appropriate therapeutical concentration of the materials, established previously in the literature [28,29,30] of 4 × MIC (125 µg/mL). Results collected in Figure 4, revealed the excellent activity of both **NT-Ag** and **NT-Ag@Ph**, which dramatically inhibited biofilm development in all *P. aeruginosa* strains. In comparison with the control, the synthesized systems were able to inhibit the development of biofilm up to 90.12% and 92.02%, respectively, for the ATCC27853 strain and 80.69% and 90.31% in the case of the clinical strain PA13. In addition, even larger inhibitions of up to 95.62% and 96.13%, respectively, were found in the case of the clinical strain PA8. Interestingly, the bare MSNs had no effect on biofilm development in strain PA13, whereas, in the other two strains, the inhibition of biofilm development was significantly lower compared to the final materials (20.29% and 13.43% for strains ATCC27853 and PA8, respectively). Therefore, it can be concluded that the observed inhibitions seem to be a consequence of the incorporation of AgCl NPs.

#### 2.2.5. Inhibition in Wound-like Medium

**Figure 5:**
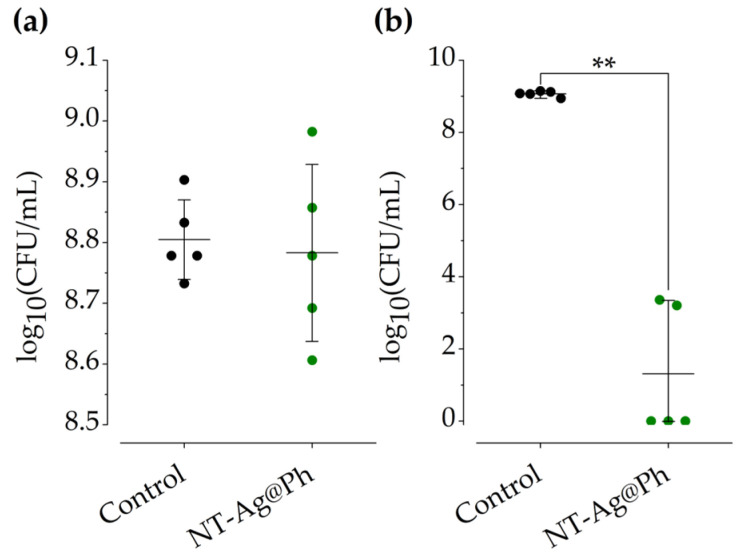
The effect of **NT-Ag@Ph** against ATCC27853 wound-like medium at 125 µg/mL (**a**) and 2 mg/mL (**b**). The control (black) and **NT-Ag@Ph** (green) are shown. ** *p* < 0.01 for Mann–Whitney test.

Inhibition was also studied in a wound-like medium. This medium composition included components that the synthesized materials would have to deal with in an in vivo model. The inhibitory effect in the wound-like medium of **NT-Ag@Ph** material was evaluated against *P. aeruginosa* ATCC27853. For this purpose, two selected concentrations of the **NT-Ag@Ph** material, i.e., 125 µg/mL (4 × MIC) and 2 mg/mL (4 × MBC), were chosen. As observed in Figure 4, bacterial growth inhibition was dose-dependent, showing no effect at concentrations of 125 µg/mL (Figure 5a) but a dramatic inhibition (99.99%) at a concentration of 2 mg/mL (Figure 5b).

#### 2.2.6. Bactericidal Mechanism of NT-Ag@Ph

**Figure 6:**
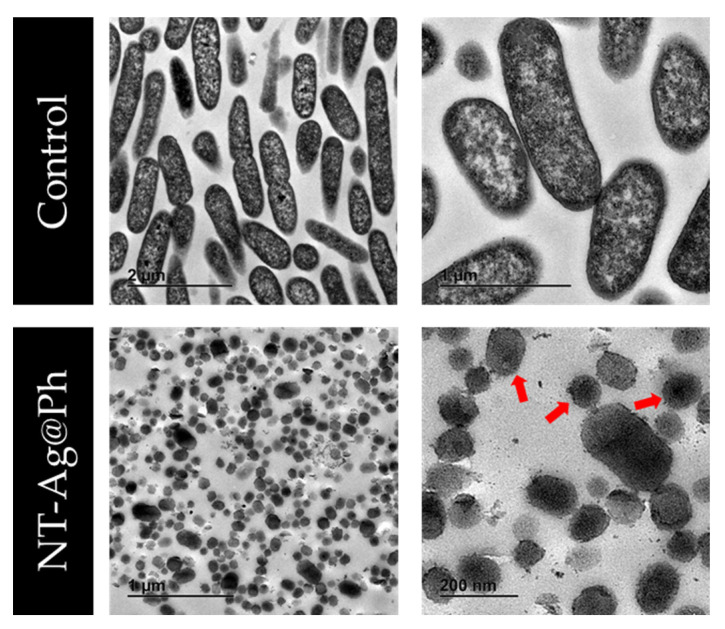
TEM images of *P. aeruginosa* ATCC27853 treated with 0 (control) and 2 mg/mL of **NT-Ag@Ph**. Red arrows indicate elements compatible with membrane vesicles.

The bactericidal mechanism of **NT-Ag@Ph** was analyzed by means of a thoughtful analysis of TEM images of *P. aeruginosa* ATCC27853 strain culture, acquired in both the absence (control) and the presence of 2 mg/mL (2 × MBC) of **NT-Ag@Ph** (Figure 6).

The control showed intact bacteria with an anodyne appearance, as well as bacilli with approximate dimensions of 1.5–2 µm long and 0.3–0.5 µm wide. In addition, in the figures, one can also observe that the cytoplasmic membrane was intimately bound to the outer membrane of the bacteria. However, TEM images acquired upon incubation of bacteria with **NT-Ag@Ph** show bacteria with clear structural and morphological changes, as only elements compatible with membranous vesicles were found. In addition to the silica nanoparticles, small nanoparticles that could be compatible with AgCl NPs were also observed.

#### 2.2.7. Cytotoxicity and Cell Proliferation Studies

**Figure 7:**
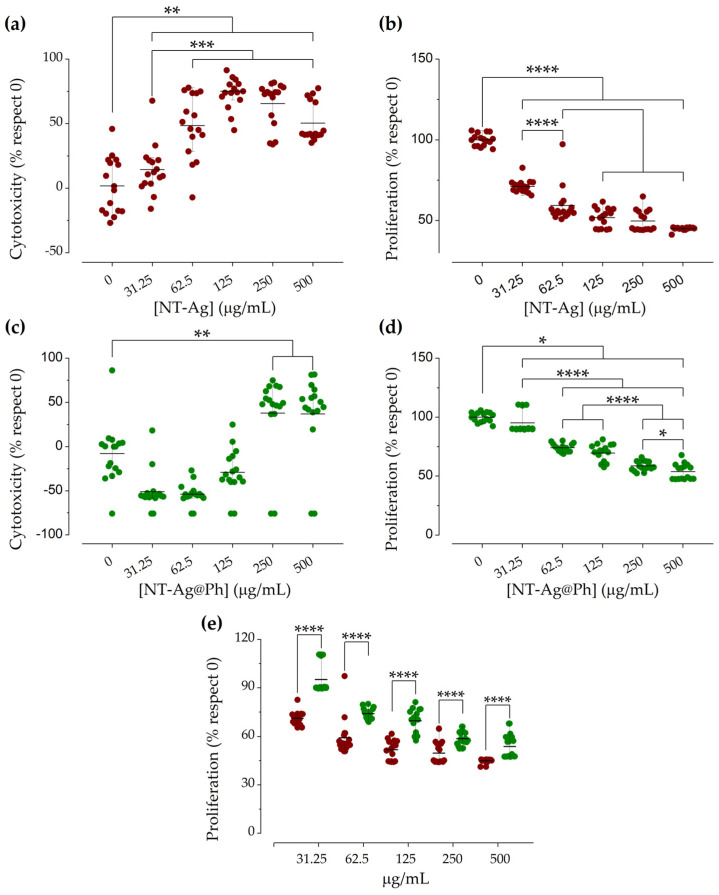
Cytotoxicity of **NT-Ag** (**a**) and **NT-Ag@Ph** (**c**), and fibroblastic proliferation of **NT-Ag** (**b**) and **NT-Ag@Ph** (**d**). Comparative proliferation (**e**) of unloaded (garnet) and loaded (green) material with phenytoin sodium. *, **, ***, **** *p* < 0.05, 0.01, 0.001, and 0.0001 for Mann–Whitney and Kruskal–Wallis tests.

The biocompatibility of **NT-Ag** and **NT-Ag@Ph** was evaluated on fibroblast fHDF/TER166. As shown in Figure 7a, **NT-Ag** was slightly cytotoxic at a concentration of 31.25 µg/mL, whereas higher cytotoxicity was observed at concentrations above 62.5 µg/mL. The material also reduced or slowed down cell proliferation in a concentration-dependent manner (Figure 7b). On the other hand, **NT-Ag@Ph** was shown to be noncytotoxic toward fibroblasts (Figure 7c) up to a concentration of 250 µg/mL, and it reduced or slowed down proliferation at above 31.25 µg/mL. Importantly, the presence of phenytoin sodium promoted fibroblast proliferation (Figure 7e), up to 27.15%, 35.75%, and 28.75% at concentrations of 31.25 µg/mL, 125 µg/mL, and 250 µg/mL, respectively. At the highest concentration tested (500 µg/mL), the effect of the encapsulated phenytoin was yet evident, but less marked (17.15%).

#### 2.2.8. Selectivity Index

According to Orme et al. [31] candidates for new drugs must have a selectivity index (SI) equal to or higher than 10, together with an MIC lower than 6.25 mg/mL (or the molar equivalent) and a low cytotoxicity. SI is used to estimate the therapeutic window of a drug and to identify drug candidates for further studies. The selectivity index (SI) of the metal complexes was calculated by dividing the IC_50_ for the fibroblast fHDF/TER166 cells (Figure S9) by the MIC for the different pathogen strains. A promising antibacterial agent is found when the SI value is higher than 10, which means that the analyzed agent is much more active against the bacteria than it is cytotoxic to the host. For the unloaded material, i.e., **NT-Ag**, the approximate IC_50_ value obtained was 151 µg/mL; therefore, the SIs obtained for each strain were below 10 (0.6, 2.41, and 4.83 for ATCC27583, PA8, and PA13, respectively) (Table S2). However, for the phenytoin-loaded system, **NT-Ag@Ph**, with MIC values of 31.25 µg/mL and IC_50_ > 500 µg/mL, SI values >16 for each of the three analyzed bacterial strains can be considered very high, highlighting **NT-Ag@Ph** as a very promising new antibacterial drug candidate.

## 3. Discussion

*P. aeruginosa* is known for its high capacity to form biofilms, which, in addition to its high resistance, is responsible for the persistence of prolonged infections in chronic wounds and burns. In this study, we fully characterized the excellent antibacterial activity, in planktonic, biofilm, and wound-healing conditions, of a novel hybrid nanosystem based on nicotinate-functionalized mesoporous silica and silver chloride nanoparticles loaded with phenytoin.

In the planktonic state, the materials synthesized in this work showed excellent antibacterial activity against the studied *P. aeruginosa* strains, with excellent MIC and MBC values lower than some studies reported with similar silver systems [32,33,34,35]. Moreover, these values were obtained with a very low content of silver, even lower than that of some commercial drugs [36]. One of the plausible reasons for this is that silver nanoparticles have different mechanisms to kill bacteria. For example, due to the affinity of silver toward thiol and phosphate or P-containing groups, the nanoparticles can interact with the outer membrane, increasing the permeability of the membrane and inducing leakage of cellular contents, leading to death of the microorganism. In addition to interaction with the membrane, inside the cells, nanoparticles can interact with intracellular components such as DNA, proteins, and ribosomes, altering structure and function. Furthermore, they can alter the respiratory chain in the inner membrane by interacting with the thiol groups of enzymes, inducing reactive oxygen species (ROS) and free radicals, generating damage to the intracellular machinery and activating the apoptosis pathway [37,38,39]. Any of these mechanisms could be responsible for the bactericidal effect observed in **NT-Ag@Ph** against the planktonic state of *P. aeruginosa* ATCC27853 strain. These results were further supported by TEM study, where no intact bacteria were observed in the presence of the material and membranous vesicles were found. Additionally, although, as observed and reported in the literature, phenytoin sodium has no antipseudomonal activity [40], our results indicate that the potential interaction between Ag^+^ and phenytoin sodium is an additive effect, because the FIC index was 0.75 and the antibacterial activities of our phenytoin-loaded materials improved those of the unloaded systems, reaching excellent values for further development of the materials as antibacterial agents.

Importantly, our phenytoin-loaded materials were able to significantly block the ability of *P. aeruginosa* to induce biofilm formation, at concentrations as low as 125–500 µg/mL (0.40–1.76 µg/mL in reference to Ag%). These values are similar to those reported with similar silver systems [41]. Nevertheless, the amount of silver in this work was 35 times lower, thus showing a higher efficiency of the final system, although these materials were not able to eradicate the biofilm, at least at the tested concentrations. Thus, using a concentration of 125 µg/mL, biofilm development was inhibited by 80–96%, which was proven to arise from the AgCl content. These results remarkably surpass those found with MSN-based materials [42,43], including those systems functionalized with silver nanoparticles and nanocomposites [41,44].

Wound-like medium, due to its composition, is more similar to what is usually found in clinical practice [45]. To the best of our knowledge, to date, MSN-based materials have not been studied in *P. aeruginosa* wound-like medium; therefore, our study focused on the use of wound-like medium, observing that, at a concentration of 2 mg/mL (4 × MBC) (6.4 µg/mL in reference to Ag%), the drug candidate synthesized in this work, namely, **NT-Ag@Ph**, showed the ability to kill 99.99% of the bacteria, a very promising result which validates the novelty and potential applicability of this nanostructured antibacterial system.

In this context, the wound-healing process includes coagulation, inflammation, proliferation, and regeneration steps. Thus, it is well known that formation of biofilms causes a persistent inflammatory state, which delays re-epithelization and wound closure. Therefore, preventing biofilm formation is of great interest for the scientific community [1]. In addition, promoting proliferation is another strategy to facilitate healing and, thus, accelerate the healing process. Therefore, to improve the wound-healing abilities of the studied systems, in the present work, almost 10% of the proliferative agent phenytoin sodium was successfully loaded into the MSN pores of the synthesized nanosystems, improving the biological properties of the studied materials.

Furthermore, in order to determine the biocompatibility of the synthesized promising nanostructured systems, a cytotoxicity and proliferation study was carried out on fHDF/TER166 fibroblasts. These cells are often used as a standardized in vitro model to study processes involving fibroblasts, such as, in this case, chronic wounds and burns. The results showed that the unloaded material was shown to be cytotoxic and to decrease cell proliferation at 31.25 µg/mL and above; however, the material containing the proliferative agent (Ph) showed better results, as it was not cytotoxic up to 250 µg/mL. Although fibroblast proliferation persisted in the presence of both **NT-Ag** and **NT-Ag@Ph**, the growth ratio decreased in both cases with respect to control. However, this decrease was lower in the case of phenytoin-loaded materials, which can be interpreted as an effect of the presence of phenytoin on a significant reduction in the silver effect on both cytotoxicity and proliferation of these cells. Thus, it might be expected that, upon increasing the amount of phenytoin drug in the **NT-Ag@Ph** material, the systems might be able to increase fibroblast proliferation. In the studied nanostructured systems, the cytotoxicity and proliferation reductions shown by the materials were because silver nanoparticles could generate ROS, thus contributing to cytotoxicity and decreasing proliferation. To the best of our knowledge, this is the first study involving a hybrid nanosystem based on MSN with a dual purpose of both preventing *P. aeruginosa* infection and promoting fibroblast proliferation without being cytotoxic. Furthermore, according to its great selective index (SI > 16), **NT-Ag@Ph** is a promising agent to be further explored for the topic preventive treatment of chronic wounds and burns.

## 4. Materials and Methods

### 4.1. General Remarks on Characterization of the Materials

For the characterization of the nanomaterials, TEM measurements were carried out on a TECNAI G2 20 TWIN operated at 200 kV and equipped with LaB6 filament and a high-angle annular dark-field scanning transmission electron microscope (HAADF-STEM). The samples for TEM were prepared by dispersion into ethanol solvent and keeping the suspension in an ultrasonic bath for 15 min; subsequently, a drop in suspension was spread onto a TEM copper grid (300 mesh) covered by a holey carbon film followed by drying at room temperature. Textural properties of the samples were obtained by N_2_ physisorption in a Tristar II Plus (Micromeritics). Samples were previously degassed in the SmartVacPrep system (Micromeritics) at 70 °C up to a vacuum level of 1 × 10^−3^ mm Hg for 12 h and finally analyzed at −196 °C. BET and BJH methods applied to the desorption branch were used to obtain the surface area and the pore size distribution. The ICP-AES study was carried out in an Agilent 5100 Dual View. Thermogravimetric analysis (TG) was performed on a TG-Q500 TA Instruments thermal analyzer starting from room temperature to 750 °C under a nitrogen atmosphere and at a heating rate of 10 °C·min^−1^. The powder XRD patterns were collected on a Phillips X’PERT powder diffractometer with CuKα radiation (λ = 1.5418 Å) in the following ranges: 0.8 < 2θ < 10° and 5 < 2θ < 90° (depending on the sample), with a step size of 0.026° and an acquisition time of 2.5 s per step at 25 °C. Fourier-transform infrared (IR) spectra (400–4000 cm^−1^) were recorded on a Nicolet FT-IR 6700 spectrometer in KBr pellets. DR-UV measurements were acquired in a UV/Vis Shimadzu spectrophotometer. These spectra were recorded at room temperature with BaSO_4_ as the reference material. ^13^C CP MAS NMR solid-state NMR measurements were conducted on powder samples and recorded, in a high-resolution mode, at 298 K on a Bruker Avance 400 WB spectrometer at 9.4 T, using 100.66 and 400.17 MHz resonance frequencies (^1^H and ^13^C, respectively). The ^13^C experiments were performed with cross-polarization (CP), high power decoupling, and magic angle spinning (MAS), using a Bruker double-bearing probe head and 4 mm zirconia rotors driven by dry air. The MAS rates were 10 kHz. The Hartmann–Hahn conditions for ^13^C were matched using adamantane. The recycle delay was 5 s, and the contact time was 2 ms. Chemical shifts were evaluated using glycine (Gly) as an external standard (dCO of Gly = 176.5 ppm).

The quantification of the absorbance in the in vitro studies was carried out using the Tecan Infinite M200 Plate Reader. The absorbances recorded were at 492 nm using 620 nm as a reference filter, 570 nm, and 600 nm. The bactericidal mechanism of the materials was observed using a Jeol JEM 1400 transmission electron microscope (Jeol Ltd., Tokyo, Japan). For this purpose, sections were collected on 200 mesh nickel grids. The Ultracut UC7 ultramicrotome (Leica) was used for sample preparation.

### 4.2. Synthesis of Mesoporous Silica Nanoparticles (MSNs)

MSN nanoparticles were synthesized by a modification of the method previously published by Zhao and coworkers [46]. A standard preparation of MSN can be summarized as follows: 2.74 mmol of CTAB (*N*-ethyltrimethylammonium bromide) was dissolved in 480 mL of water; subsequently, NaOH (2.0 M, 3.5 mL) was added at room temperature, and the mixture was heated up to 80 °C. Then, 5 mL (22.4 mmol) of the silica precursor TEOS (tetraethyl orthosilicate) was added dropwise and left under vigorous stirring for 2 h. The white precipitate was filtered and washed with abundant water and methanol and dried for 24 h at 80 °C in an oven. Finally, the sample was calcined at 550 °C for 24 h.

### 4.3. Functionalization of Silica Materials with Amino Ligand: Synthesis of MSN-AP

The functionalization of the MSN with 3-aminopropyltriethoxysilane was carried out using a 1:2 MSN:AP mass ratio. For this purpose, the MSN was dehydrated under vacuum at 80 °C for 24 h. After that, the material was suspended in dry toluene (50 mL). Once the material was dispersed, the amino ligand was added, and the mixture was heated at 110 °C and refluxed for 48 h. After this time, the suspension was centrifuged (10 min, 6000 rpm); then, the solid was washed with abundant toluene and diethyl ether, and dried on a stove. The material obtained was named **MSN-AP**.

### 4.4. Preparation of MSN-AP-NT

MSP-AP was functionalized with the theoretical level of 10% *w*/*w* SiO_2_/NT. For the incorporation of the nicotinic ligand, an EDAC coupling process in the MES buffer was carried out. Briefly, a solution of 2-(allylthio)nicotinic acid (NT) in DMSO was added to the MES buffer with EDAC and NHS in a molar proportion of 1:2.5. The mixture was kept under vigorous stirring for 15 min at room temperature. After that, **MSN-AP** was added, and the resulting solution was maintained under stirring for an additional 2 h at room temperature. Finally, the solid was isolated by centrifugation (6000 rpm, 10 min) and washed with DMSO, water, ethanol, and diethyl ether to give the material **MSN-AP-NT**.

### 4.5. Preparation of NT-Ag and NT-Ag@Ph

A suspension of **MSN-AP-NT** (250 mg) in 55 mL of acetonitrile was stirred at 80 °C; subsequently, 36 µL of trimethylamine (0.256 mmol) and 21.74 mg (0.128 mmol) of silver nitrate (theoretical level of 3.4% Ag/SiO_2_) were added. After that, the mixture was heated up to 90 °C and refluxed in the darkness for 24 h. The solid product was isolated by centrifugation (6000 rpm, 10 min) and washed several times with acetonitrile and water. The product was dried for 12 h at 80 °C. The material obtained was named **NT-Ag**.

To load the **NT-Ag** material with phenytoin sodium (Ph), a 1 mL saturated solution of the drug in water was added to 10 mg of the **NT-Ag** material. This loaded material was left at 500 rpm and 2 °C for 20 h. The solid product was isolated by centrifugation (6000 rpm, 10 min) and washed one time with water/EtOH 1:1. The product was dried at room temperature. The phenytoin sodium (Ph)-loaded material was named **NT-Ag@Ph**.

### 4.6. In Vitro Studies

#### 4.6.1. Bacteria

Three different *P. aeruginosa* strains related to SSI were studied (Table 5): an American Culture Collection (ATCC) strain (ATCC27853) and two clinical strains isolated in the Clinical Microbiology department of the Fundación Jimenez Díaz (PA8 isolated from a 39 year old man with an infected wound and PA13 isolated from an 80 year old man with an infected wound). All strains were stored frozen at −80 °C until experiments were performed.

#### 4.6.2. Synergy Evaluation between Silver Nitrate and Phenytoin Sodium

The interaction type between silver nitrate and phenytoin sodium against *P. aeruginosa* in planktonic form was studied using the checkerboard method [26,27]. The interaction between silver nitrate and phenytoin sodium can be quantified by the fractional inhibitory concentration (FIC). For two antibacterial compounds, A and B, acting individually or in combination,

$$\mathrm{FIC}\;\mathrm{index}=\frac{A}{{\mathrm{MIC}}_{A}}+\frac{B}{{\mathrm{MIC}}_{B}},$$

where A is the MIC of silver nitrate, and B is the MIC of phenytoin sodium in combination, while MIC_A_ and MIC_B_ are the MIC of AgNO_3_ and phenytoin sodium, respectively. The FIC index is the sum of FIC_A_ and FIC_B_. An FIC index of <0.5 indicates synergism, >0.5–1 indicates an additive effect, >1 but <4 indicates indifference, and ≥4 indicates antagonism [27].

Briefly, using a 96-well plate, a twofold dilution of freshly prepared AgNO_3_ and phenytoin sodium at different concentrations in MHB was dispensed in a checkerboard array and inoculated with 10^5^ CFU/mL of the bacterial suspension. One well with no antibiotics was used as the positive control. It was incubated for 18 h at 37 °C and 5% CO_2_; after incubation, bacterial viability was determined by the addition of 20 µL of 5 mg/mL of 3-(4,5-dimethylthiazol-2-yl)-2,5-diphenyltetrazolium bromide (MTT) (Sigma Aldrich, Merck, Darmstadt, Germany) and incubating for 1 h at 37 °C and 5% CO_2_. This experiment was performed in duplicate, and the mean was presented.

#### 4.6.3. Minimum Inhibitory Concentration and Minimum Bactericidal Concentration

The minimum inhibitory concentrations (MIC) of the unloaded and phenytoin sodium (Ph)-loaded hybrid nanosystems were determined according to the standardized Clinical and Laboratory Standards Institute Microdilution method [47]. The inoculum was prepared from overnight cultures with a cell concentration of 1–5 × 10^8^ colony-forming units per mL (CFU/mL) corresponding to 0.5 on the McFarland scale. It was then diluted 1:100 in Mueller–Hinton Broth (MHB) medium. In a 96-well microplate, serial dilutions were performed from 2000 to 1.9531 µg/mL, and then the inoculum was added at the concentration of 1–5 × 10^6^ CFU/mL. Finally, it was incubated for 18 h at 37 °C and 5% CO_2_ and developed using MTT (3-(4,5-dimethyl-2-thiazolyl)-2,5-diphenyltetrazole bromide)) as the developing agent. The MIC was defined as the lowest concentration where no microbial growth was observed after 18 h of incubation.

The minimum bactericidal concentration (MBC) was determined using the Flash microdilution method described above with some modifications [48]. Briefly, 20 μL of each well after 24 h of incubation was mixed with 180 μL of Tryptic Soy Broth (TSB) broth in a new 96-well plate and statically incubated at 37 °C and 5% CO_2_ for 24 h. The MBC was defined as the bactericidal concentration of each well after 24 h incubation, measuring the absorbance at 600 nm. The MBC was defined as the lowest concentration where the death of 99.9% of the initial inoculum occurred.

#### 4.6.4. Minimal Biofilm Inhibitory Concentration and Minimal Biofilm Eradication Concentration

The minimum inhibitory concentration (MBIC) and minimum biofilm eradication concentration (MBEC) were determined using the methodology previously described [49]. The MBIC is the minimum concentration necessary to inhibit the visible growth of a bacterial biofilm. To determine the MBIC, biofilm formation was induced on the pegs of the Calgary device. For this purpose, a 96-well plate (Thermo Fisher Scientific, Waltham, MA, USA) was inoculated with 200 µL of TSB containing 10^6^ CFU/mL bacteria per well. The lid (Thermo Fisher Scientific) of the Calgary device was then placed on and the plate was incubated at 37 °C and 5% CO_2_ for 24 h. After incubation, the lid pegs were rinsed twice using wells containing 200 µL of 0.9% NaCl saline. The lid was then placed on a plate with different concentrations of the tested materials, starting at 1000 to 1.953 µg/mL, with a twofold dilution; MHB was added to a final volume of 200 µL per well. This plate was incubated at 37 °C and 5% CO_2_ for at least 20 h. After incubation, MBIC values were determined using MTT as the developing agent. For this purpose, 20 µL of MTT was added to each well and kept shaking at 90 rpm in an incubator at 37 °C for 1.5 h; after incubation, absorbance was measured at 570 nm. The experiments were performed in triplicate.

MBEC is the minimum concentration necessary to kill a bacterial biofilm. For MBEC, the MBIC lid was rinsed twice in a plate with wells containing 200 µL of 0.9% NaCl saline, placed in a plate with 200 µL of TSB, and statically incubated at 37 °C and 5% CO_2_ for 24 h. After incubation, MBEC was determined by measuring absorbance at 600 nm.

#### 4.6.5. Effect on Biofilm Development

To study whether the materials inhibit biofilm development, one 96-well plate was used for each *P. aeruginosa* strain. The materials used were unmodified MSN as control and **NT-Ag** loaded and unloaded with phenytoin sodium (**NT-Ag@Ph** and **NT-Ag**). The concentration used for each material was four times the MIC of the drug-containing material, considering previous studies stating that 4 × MIC is a good therapeutic approach [28,29,30]. For this purpose, 200 µL of 1 × 10^6^ CFU/mL in TSB + 1% glucose of each strain with a 4 × MIC concentration of the material was deposited in a 96-well flat-bottom plate and incubated at 37 °C and 5% CO_2_ for 24 h. After incubation, each well was rinsed twice with 200 µL of saline. Biofilm quantification was performed according to a previously reported methodology [50]. To begin with, each well was fixed with 200 µL of MeOH and kept for 20 min in open air. The supernatant was then removed and allowed to dry at 60 °C. After fixation, staining was performed. For this purpose, 150 µL of 1% safranin was added for 15 min to allow the dye to penetrate through the biofilm. Then, each well was rinsed twice with 200 µL of distilled water and it was solubilized with absolute ethanol. Finally, absorbance was measured at 492 nm in a colorimeter using 620 nm as a reference filter. This experiment was performed in eight wells for each material and strain and in triplicate (*n* = 24).

#### 4.6.6. Inhibition in Wound-Like Medium

Biofilm development in a wound-like medium was determined using previously described methods based on the Lubbock chronic wound medium [45,51]. The wound-like medium was composed of 50% bovine plasma (Sigma-Aldrich, St. Louis, MO, USA), 45% Bolton broth (Sigma-Aldrich, St. Louis, MO, USA) and 5% lacquered horse red blood cells (Thermo Fisher Scientific), supplemented with or without (positive control) the tested material at a concentration of 4 × MIC or 4 × MBC. Half a milliliter of each medium was incubated with 5 µL of 10^8^ CFU/mL of *P. aeruginosa* strain ATCC27853 for 24 h at 37 °C and 5% CO_2_. After incubation, 0.5 mL of saline was added and sonicated for 5 min. Then, this sonicated saline was serially diluted with saline, and the CFU per milliliter was estimated using the drop plate method on MacConkey agar plates. This experiment was performed in quintuplicate for each material (*n* = 5).

#### 4.6.7. Bactericidal Mechanism of the NT-Ag@Ph Material Using TEM

The bactericidal mechanism of the final **NT-Ag@Ph** material in strain ATCC27853 was analyzed by transmission electron microscopy (TEM). The concentration of material used was 2 × MBC. The protocol used for this was described previously [52]. Thin sections (60 nm) were cut for TEM of the resin-included bacteria using a Leica Ultracut ultramicrotome UC7 (Leica). Sections were collected on 200 mesh nickel grids and examined using a Jeol JEM 1400 transmission mission electron microscope (Jeol Ltd., Tokyo, Japan).

#### 4.6.8. Cytotoxicity and Cell Proliferation

The biocompatibility of **NT-Ag** and **NT-Ag@Ph** materials were tested in fHDF/TER166 fibroblast cell line (Evecyte, Vienna, Austria). For this purpose, the range of material concentrations used was 31.25–500 µg/mL. The cytotoxicity study was conducted using a CytoTox 96^®^ NonRadioactive Cytotoxicity Assay (Promega, Madison, WI, USA), according to a previously published methodology, after 48 h of incubation.

For the cell proliferation study, cells from the fibroblast cell line fHDF/TER166 were seeded in a concentration of 10,000 cells/cm^2^ on 96-well plates in DMEM with 10% fetal bovine serum and 1% penicillin–streptomycin (DMEM, Invitrogen, Thermo Fisher Scientific Inc., Waltham, MA, USA). Then, the cells were incubated at 37 °C and 5% CO_2_ overnight. Thereafter, the supernatant was removed, and cells were incubated in the presence of serial dilutions of **NT-Ag** or **NT-Ag@Ph** from 31.25 to 500 µg/mL for 48 h (*n* = 8 per concentration). After incubation, cell proliferation was determined by adding 20 µL of 5 mg/mL of MTT and incubating for 3 h at 37 °C and 5% CO_2_. This experiment was performed in duplicate.

#### 4.6.9. Statistical Analysis and IC_50_ Assay

Statistical analyses were performed using GraphPad Prism 8 software (version 8.01). Data were evaluated using a two-sided Mann–Whitney nonparametric test to compare two groups and a Kruskal–Wallis nonparametric test to compare more than two groups. Statistical significance was set at *p*-values ≤0.05. All results are represented as the median and interquartile range. The IC_50_ studies were carried out using AAT Bioquest, Inc. (*Quest Graph™ IC50 Calculator*). AAT Bioquest. https://www.aatbio.com/tools/ic50-calculator (accessed on 3 June 2022).

#### 4.6.10. Ph Release Studies

Ph release of the **NT-Ag@Ph** material was carried out in order to determine the stability of the system and the quantity of Ph in the solution at different times. For this, 1 mg of **NT-Ag@Ph** material was dispersed in 1 mL of a 95:5 mixture of PBS buffer at pH = 7.4 and DMSO, respectively. The mixture was incubated at different times at 37 °C with slight shaking (30 rpm) using an incubator Roto-587 Therm (Benchmark) with control of the temperature. The suspensions were then filtered through a 0.2 µm nylon filter, and the suspension was analyzed by HPLC (Flexar Perkin-Elmer) using a C_18_ column (COSMOSIL 5C_18_-MS-II 4.6 mm I.D. × 250 mm with a particle size of 4.4 µm) and a mobile phase composed of 60:40 MeOH:H_2_O at 0.7 mL/min. Ph appeared in the chromatograms at a retention time of 9.2 min.

## 5. Conclusions

The **NT-Ag@Ph** material was shown to be a promising nanomaterial against *P. aeruginosa* strains, including the two clinical strains and, in particular, the PA13 strain, being able to inhibit and eradicate *P. aeruginosa* in the planktonic form, as well as in the biofilm state. Although the material was not able to eradicate the *P. aeruginosa* biofilm, the hybrid nanosystem based on nicotinate-functionalized MSN and AgCl NPs loaded with phenytoin inhibited the development of these biofilms and was able to inhibit in wound-like medium when used at high concentrations. Even though the final material significantly reduced fibroblast proliferation, it was able to inhibit biofilm development in all three *P. aeruginosa* strains without showing cytotoxicity, resulting in a high selectivity and pointing toward the highly promising applicability of this system in chronic wounds and burns.

## Figures and Tables

**Table 1:** Textural parameters of selected MSN materials obtained from nitrogen adsorption–desorption analysis.

Material	BET Surface (m^2^/g)	Pore Volume (cm^3^/g)	Pore Diameter (nm)
**MSN**	1102	0.75	2.84
**NT-Ag**	115	0.18	<2.00
**NT-Ag@Ph**	48	0.07	<2.00

**Table 2:** Weight percentage and functionalization/encapsulation ratio of AP, NT, Ph, and Ag in the materials synthesized.

Material	%AP	%NT	%Ph	% Experimental Ag	mmol of NT, Ph or Ag per g of Material
**MSN**	8.68	-	-	-	-
**MSN-AP-NT**	8.68	2.52	-	-	0.14 (NT)
**NT-Ag**	8.68	2.52	-	0.35	0.03 (Ag)
**NT-Ag@Ph**	8.68	2.52	9.30	0.32	0.37 (Ph); 0.03 (Ag)

**Table 3:** Minimum inhibitory concentration (MIC) and minimum bactericidal concentration (MBC) for **NT-Ag** and **NT-Ag@Ph** against three different planktonic *P. aeruginosa* strains. Data are given in µg/mL of material, and data in parentheses refer to the µg/mL of silver.

Material	ATCC27853		PA8		PA13	
	MIC	MBC	MIC	MBC	MIC	MBC
**NT-Ag**	250 (0.88)	500 (1.76)	62.50 (0.22)	125 (0.44)	31.25 (0.11)	250 (0.88)
**NT-Ag @Ph**	31.25 (0.10)	500 (1.60)	31.25 (0.10)	125 (0.40)	31.25 (0.10)	250 (0.80)

**Table 4:** Minimum biofilm inhibitory concentration (MBIC) and minimal biofilm eradication concentration (MBEC) for **NT-Ag** and **NT-Ag@Ph** against three different *P. aeruginosa* strains. Data are given in µg/mL of material, and data in brackets refer to the µg/mL of silver.

Material	ATCC27853		PA8		PA13	
	MBIC	MBEC	MBIC	MBEC	MBIC	MBE
**NT-Ag**	250 (0.88)	>1000 (3.51)	125 (0.44)	>1000 (3.51)	500 (1.76)	>1000 (3.51)
**NT-Ag@Ph**	125 (0.40)	>1000 (3.20)	125 (0.40)	1000 (3.20)	500 (1.60)	>1000 (3.20)

**Table 5:** Antibiotic susceptibility pattern of the three *P. aeruginosa* studied in this work. Data are given in mg/L. S: susceptible; R: resistant.

Antibiotic	ATCC27853	PA8	PA13
Amikacin	S (2)	S (2)	S (2)
Aztreonam	S (2)	S (4)	S (2)
Cefepime	S (2)	S (1)	R (16)
Ceftazidime	S (2)	S (2)	S (8)
Ceftolozane/tazobactam	S (0.5)	S (0.5)	S (1)
Ciprofloxacin	S (<0.25)	S (≤0.25)	R (≥4)
Colistin	S (<0.5)	S (≤0.5)	S (≤0.5)
Gentamycin	S (<1)	S (≤1)	S (≤1)
Imipenem	S (2)	S (1)	S (1)
Meropenem	S (<0.25)	S (≤0.25)	S (1)
Piperacillin/tazobactam	S (<4)	S (≤4)	R (≥128)
Tobramycin	S (<1)	S (≤1)	S (≤1)

## Data Availability

Data is contained within the article and Supplementary Materials.

## Supplementary Materials

The following supporting information can be downloaded at https://www.mdpi.com/article/10.3390/ph15070884/s1: Figure S1. Particle size distributions of **NT-Ag** (**a**) and **NT-Ag@Ph** (**b**); Figure S2. Nitrogen adsorption (solid line) and desorption (dashed line) isotherms (**a**) and pore size distribution (**b**); Figure S3. Thermogravimetric studies of the functionalized materials, Figure S4. XRD diffractograms of **NT-Ag** (**a**) and **NT-Ag@Ph** (**b**) with AgCl and MSN@Ph, respectively; Figure S5. FT-IR spectra of the starting material MSN and the final functionalized materials; Figure S6. DR-UV spectra of the final materials; Figure S7. ^13^C CP MAS NMR spectra of **NT-Ag@Ph**; Figure S8. Ph release studies; Figure S9. IC_50_ assay of **NT-Ag** (green) and **NT-Ag@Ph** (garnet); Table S1. XRD data of MSN and its functionalized materials; Table S2. IC_50_, MIC and selectivity index of **NT-Ag** and **NT-Ag@Ph**.
