# Supplementary material for: Hybrid Nanosystems Based on Nicotinate-Functionalized Mesoporous Silica and Silver Chloride Nanoparticles Loaded with Phenytoin for Preventing Pseudomonas aeruginosa Biofilm Development
Source: Pharmaceuticals (Basel). 2022 Jul 18;15(7):884. doi: 10.3390/ph15070884 (PMC9316646; doi:10.3390/ph15070884)
Supplement: Supplementary file 1 [file pharmaceuticals-15-00884-s001.zip › pharmaceuticals-1803391-supplementary.pdf]

SUPPORTING INFORMATION

Hybrid Nanosystems Based onNicotinate-Functionalized Mesoporous Silica and Silver Chloride Nanoparticles Loaded with Phenytoin for Preventing *Pseudomonas aeruginosa* Biofilm Development

2.1. Physicochemical characterization of functionalized NPs.

1. Particle size distribution of **NT-Ag** and **NT-Ag@Ph**
2. Textural properties. Nitrogen adsorption-desorption isotherms and pore size distribution of MSN and the final materials **NT-Ag** and **NT-Ag@Ph**.
3. TG of the functionalized materials.
4. Characterization by powder X-ray diffraction studies.
5. FT-IR and UV-Vis spectroscopy
6. Solid State RMN

2.2. In Vitro Studies of antibacterial activity

1. Ph release studies
2. IC50 assay and Selectivity Index

2.1. Physicochemical characterization of functionalized NPs.

1. Particle size distribution of **NT-Ag** and **NT-Ag@Ph**

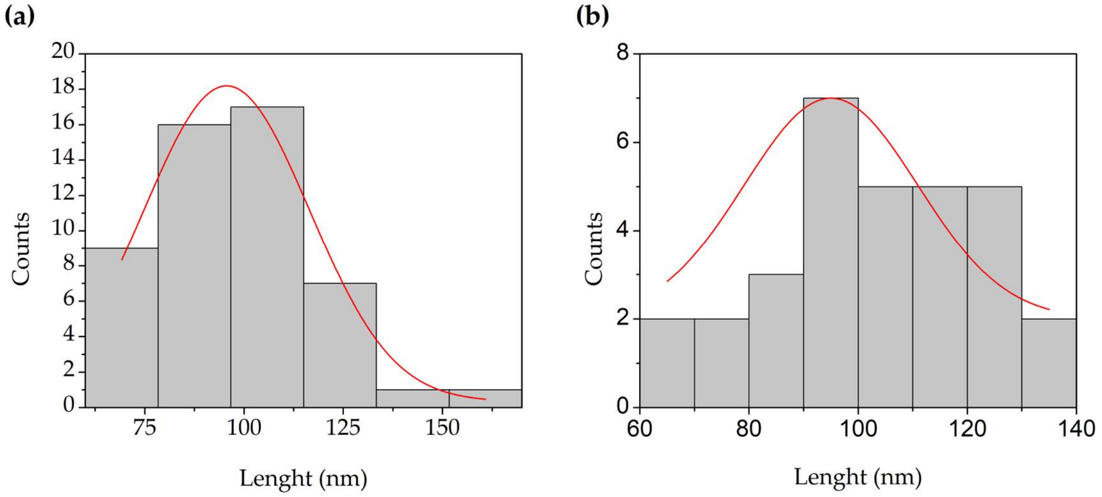

Figure S1. Particle size distributions of **NT-Ag** (**a**) and **NT-Ag@Ph** (**b**).

2. Nitrogen adsorption-desorption isotherms and pore size distribution

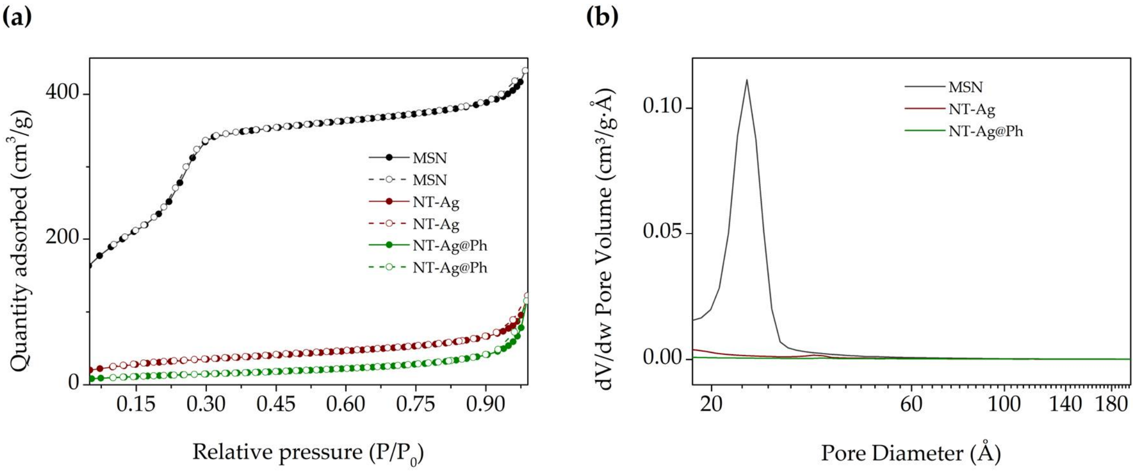

Figure S2. Nitrogen adsorption (solid line) and desorption (dashed line) isotherms (**a**) and pore size distribution (**b**).

3. TG of the functionalized materials

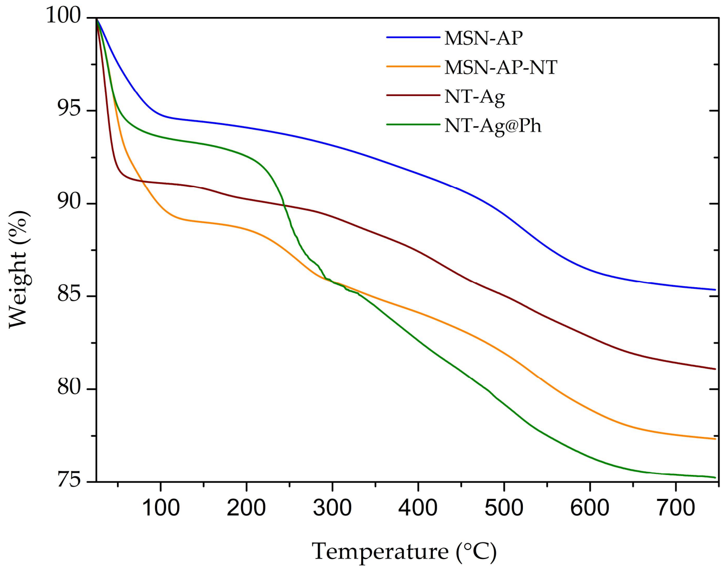

Figure S3. Thermogravimetric studies of the functionalized materials.

4. Powder X-ray diffraction

Table S1. XRD data of MSN and its functionalized materials.

Material	*hkl*	2θ(°)	d_{hkl} (Å)	a_0 (Å)
	100	2.47	35.67	41.19
**MSN**	110	4.12	21.44	24.76
	200	4.74	18.63	21.51
**MSN-AP**	100	2.50	35.32	40.78
**MSN-AP-NT**	100	2.66	33.23	38.37
**NT-Ag**	100	2.66	33.23	38.37
**NT-Ag@Ph**	100	-	-	-

Below (Figure S4), the large angle diffractograms of the materials synthesized in this work are found. The diffractogram (Figure S4a) showed eight diffraction peaks around 2θ 27.8, 32.2, 46.2, 54.8, 57.5, 67.5; 76.7 and 85.7°. It was observed using the PDF® database of the International Center for Diffraction Data that the **NT-Ag** diffractogram matched the diffractogram of AgCl, so it can be said that these nanoparticles are AgCl.

On the other hand, the correct encapsulation of phenytoin sodium in the pores of MSN was also confirmed by X-ray diffractogram study up to 50º (Figure S4b). The diffractogram of **NT-Ag@Ph** material showed various peaks corresponding to the drug, which was compared with a diffractogram of the starting material with phenytoin sodium.

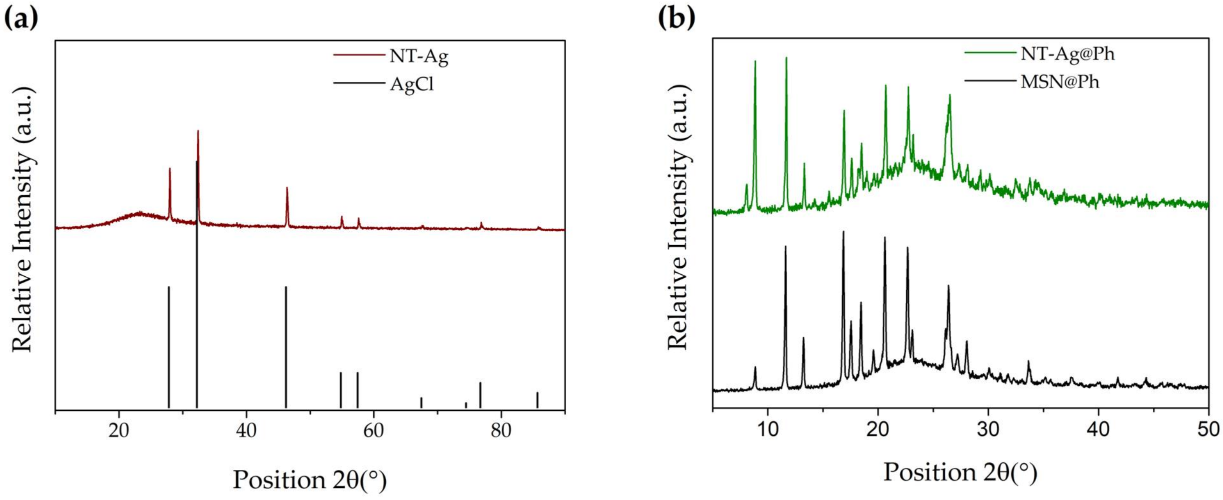

Figure S4. XRD diffractograms of **NT-Ag** (**a**) and **NT-Ag@Ph** (**b**) with AgCl and MSN@Ph, respectively.

5. FT-IR and UV-Vis spectroscopy

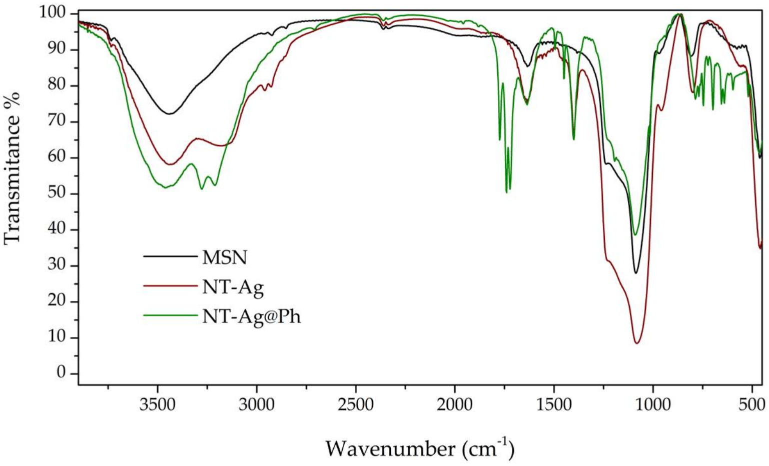

Figure S5. FT-IR spectra of the starting material **MSN** and the final functionalized materials.

The final materials **NT-Ag** and **NT-Ag@Ph** were characterized by infrared (IR) and diffuse reflectance ultraviolet spectroscopy (DR-UV). In the case of infrared spectra (Figure S5) in the starting and final materials, a broadband corresponding to the siloxane group to the siloxane group (Si-O-Si) can be observed at approximately 1100 cm^{-1} and the vibration band of the Si-O bonds, which appears at about 900 cm^{-1}. Also, the spectra show, the typical O-H vibration bands attributed to the silanol groups and the adsorbed water molecules (between 3500 and 3200 cm^{-1}) and the band corresponding to the deformation vibrations of the water molecules adsorbed on the surface of the material at ca 1625 cm^{-1}. In addition to the signals corresponding to **MSN**, some bands of relatively low intensity are visible in the spectra of unloaded material corresponding to the NT ligand; thus, C-H and N-H vibrational bands were recorded between 2700 and 3400 cm^{-1}, and the C=C and COO or CON signals were observed between approximately 1350 and

1750 cm^{-1}. Moreover, the **NT-Ag@Ph** loaded material shows, in addition to the signals mentioned above, a set of signals with higher intensity corresponding to phenytoin sodium encapsulated. These signals are visible between 3200 and 3400 cm^{-1} (corresponding to the N-H bonds), 1720 and 1770 cm^{-1} (attributed to the C=O bonds) and finally between 700-800 cm^{-1} (C-H bending signals).

In the DR-UV spectra (Figure S6), an intense peak at 220 nm and a lower intensity shoulder around 270 nm can be observed, due to the incorporation of the NT ligand and phenytoin sodium. Also, in both spectra, an intense peak is observed at 400 nm, which corresponds to the surface plasmon resonance of silver nanoparticles.

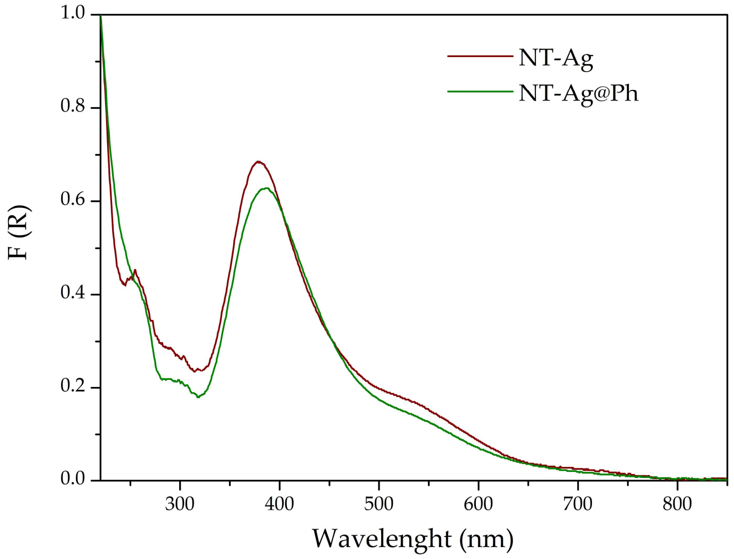

Figure S6. DR-UV spectra of the final materials.

6. Solid-state NMR spectroscopy

The **NT-Ag@Ph** material was characterized by ^{13}C CP MAS spectroscopy (Figure S7). The spectrum shows the signals corresponding to the aliphatic carbons of AP fragment (Si-CH_2-CH_2-CH_2-N) at 12, 26 and 44 ppm. In addition, at around 75 ppm the signal of the C-N carbon of phenytoin sodium can be observed. Furthermore, a cluster of signals is observed between 130 and 145 ppm due to the aromatic carbons of the NT ligand and the drug. Finally, the spectrum shows between 160 and 193 signals corresponding to the C=O and C-N groups of NT and phenytoin sodium.

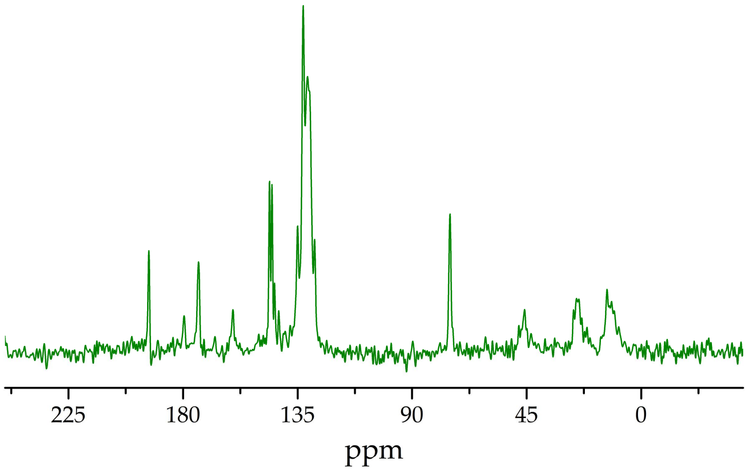

Figure S7. ^{13}C CP MAS NMR spectra of **NT-Ag@Ph**.

2.2. In Vitro Studies of antibacterial activity

1. Release Studies

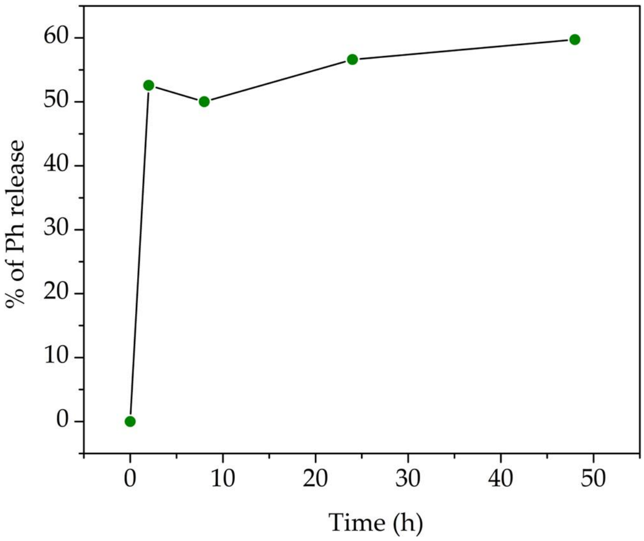

Figure S8. Release Studies

2. IC50 assay and Selectivity Index

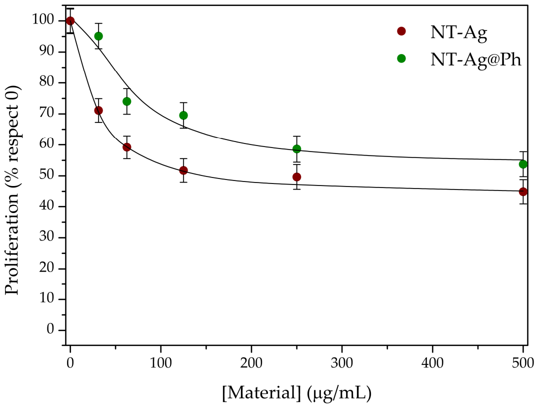

Figure S9. IC50 assay of **NT-Ag** (green) and **NT-Ag@Ph** (garnet).

Table S2. IC50, MIC and selectivitiy index of **NT-Ag** and **NT-Ag@Ph**.

Material	IC50 (μg/mL)	MIC (μg/mL)			Selectivity index		
		ATCC27853	PA8	PA13	ATCC27853	PA8	PA13
**NT-Ag**	151	250	62.5	31.25	0.60	2.42	4.83
**NT-Ag@Ph**	>500	31.25	31.25	31.25	>16	>16	>16
